# Plasmid Dynamics in KPC-Positive *Klebsiella pneumoniae* during Long-Term Patient Colonization

**DOI:** 10.1128/mBio.00742-16

**Published:** 2016-06-28

**Authors:** Sean Conlan, Morgan Park, Clayton Deming, Pamela J. Thomas, Alice C. Young, Holly Coleman, Christina Sison, NISC Comparative Sequencing Program, Rebecca A. Weingarten, Anna F. Lau, John P. Dekker, Tara N. Palmore, Karen M. Frank, Julia A. Segre

**Affiliations:** a National Human Genome Research Institute, Bethesda, Maryland, USA; b National Institutes of Health Intramural Sequencing Center (NISC), Rockville, Maryland, USA; c National Institutes of Health Clinical Center, Bethesda, Maryland, USA

## Abstract

Carbapenem-resistant *Klebsiella pneumoniae* strains are formidable hospital pathogens that pose a serious threat to patients around the globe due to a rising incidence in health care facilities, high mortality rates associated with infection, and potential to spread antibiotic resistance to other bacterial species, such as *Escherichia coli*. Over 6 months in 2011, 17 patients at the National Institutes of Health (NIH) Clinical Center became colonized with a highly virulent, transmissible carbapenem-resistant strain of *K. pneumoniae*. Our real-time genomic sequencing tracked patient-to-patient routes of transmission and informed epidemiologists’ actions to monitor and control this outbreak. Two of these patients remained colonized with carbapenemase-producing organisms for at least 2 to 4 years, providing the opportunity to undertake a focused genomic study of long-term colonization with antibiotic-resistant bacteria. Whole-genome sequencing studies shed light on the underlying complex microbial colonization, including mixed or evolving bacterial populations and gain or loss of plasmids. Isolates from NIH patient 15 showed complex plasmid rearrangements, leaving the chromosome and the *bla*_KPC_-carrying plasmid intact but rearranging the two other plasmids of this outbreak strain. NIH patient 16 has shown continuous colonization with *bla*_KPC_-positive organisms across multiple time points spanning 2011 to 2015. Genomic studies defined a complex pattern of succession and plasmid transmission across two different *K. pneumoniae* sequence types and an *E. coli* isolate. These findings demonstrate the utility of genomic methods for understanding strain succession, genome plasticity, and long-term carriage of antibiotic-resistant organisms.

## IMPORTANCE

In 2011, the NIH Clinical Center had a nosocomial outbreak involving 19 patients who became colonized or infected with *bla*_KPC_-positive *Klebsiella pneumoniae*. Patients who have intestinal colonization with *bla*_KPC_-positive *K. pneumoniae* are at risk for developing infections that are difficult or nearly impossible to treat with existing antibiotic options. Two of those patients remained colonized with *bla*_KPC_-positive *Klebsiella pneumoniae* for over a year, leading to the initiation of a detailed genomic analysis exploring mixed colonization, plasmid recombination, and plasmid diversification. Whole-genome sequence analysis identified a variety of changes, both subtle and large, in the *bla*_KPC_-positive organisms. Long-term colonization of patients with *bla*_KPC_-positive *Klebsiella pneumoniae* creates new opportunities for horizontal gene transfer of plasmids encoding antibiotic resistance genes and poses complications for the delivery of health care.

## INTRODUCTION

Multidrug-resistant organisms are widely recognized as a serious threat to the delivery of health care. Public health authorities have sounded the alarm in Europe, the United Kingdom, and the United States to rein in the unnecessary use of antibiotics, to stimulate the development of new classes of antibiotics, and to make infection control a high priority (1, 2). Recent years have seen a rise of worrisome Gram-negative bacteria producing carbapenemase enzymes—which hydrolyze carbapenem antibiotics and are frequently resistant to multiple classes of antibiotics.

First detected in *Klebsiella pneumoniae*, the *Klebsiella pneumoniae* carbapenemase (KPC) enzyme is typically plasmid encoded*.* A major public health concern is whether plasmid-encoded antibiotic resistance genes will move, through horizontal gene transfer, into strains associated with more common infections. Carbapenemase-producing organisms (CPOs) harboring plasmid-encoded *bla*_KPC_ include *Klebsiella pneumoniae*, *Klebsiella oxytoca*, *Escherichia coli*, *Enterobacter cloacae*, *Citrobacter freundii*, and *Pantoea* species, as well as some non-*Enterobacteriacae* species (3–5). Because of their prevalence and ability to spread antibiotic resistance to other common human-associated strains, these CPOs have been referred to by public health experts around the world as “nightmare bacteria” (6) or as a “catastrophic threat” (7) whose spread could lead to “antibiotic Armageddon” (8).

Patients who have gastrointestinal tract colonization with *bla*_KPC_-positive (*bla*_KPC_^+^) *K. pneumoniae* strains are at risk for developing infections that are difficult or nearly impossible to treat with existing antibiotic options. Colonized patients also serve as reservoirs for transmission; early detection of carriage enables health care facilities to contain spread through patient isolation and other measures. Multidrug-resistant-organism colonization complicates transfer of patients between health care facilities and burdens infection control resources (9), particularly because many patients who acquire multidrug-resistant Gram-negative bacterial colonization develop prolonged carriage and may serve as reservoirs for transmission over an extended time (10). Longer duration of carriage is associated with antibiotic use (11), repeated hospitalization, indwelling medical devices, and low functional status (12)—all characteristics of chronically poor health. Prolonged stays in intensive care units have also been linked to the development of ultra-low-diversity communities of multidrug-resistant organisms in some patients (13). Recent reports have shown that patients can remain colonized with *bla*_KPC_^+^ *K. pneumoniae* for months to years after detection of the first positive culture (10, 12).

Typically, carriage is monitored by culture-based techniques and *bla*_KPC_ PCR. While these characterizations provide the necessary clinical information for patient treatment and hospital infection control, they do not provide the resolution required to unravel underlying biological and microbial genomic changes. For example, mixed populations are difficult to detect in the absence of morphological differences. Likewise, it is often difficult to detect genomic rearrangements in chromosome or plasmids that might alter bacterial fitness and the antibiotic resistance trajectory (10, 12). Studies with finer resolution may reveal how these antibiotic-resistant organisms adapt to a human host and how plasmids diversify, possibly leading to horizontal gene transfer of antibiotic resistance genes to other organisms.

In 2011, the National Institutes of Health (NIH) Clinical Center, a 240-bed research hospital, experienced a nosocomial outbreak in which 19 patients developed colonization or infection with *bla*_KPC_^+^ *Klebsiella pneumoniae*. Genomic sequencing was used to impute a transmission map for a clonal cluster belonging to the dominant sequence type (ST), ST258 (14). Further analysis using single-molecule real-time (SMRT) sequencing (15) determined that the clone transmitted in the outbreak contained three plasmids that were maintained over the course of the outbreak, including the *bla*_KPC_-carrying pKpQIL plasmid (5). During care at our institution over 2 to 3 years, persistent carriage was detected in two subjects from the 2011 cluster. Whole-genome sequence analysis of isolates from these two individuals identified a variety of changes, both subtle and large, in the *bla*_KPC_^+^ organisms. Here we provide results of detailed genomic analysis demonstrating unrecognized mixed colonization, plasmid recombination, and plasmid diversification.

## RESULTS

### Long-term outcomes of the 2011 *bla*_KPC_^+^ *K. pneumoniae* patient cluster.

Among the 19 patients who were part of the 2011 outbreak, 7 patients died from *bla*_KPC_^+^ *K. pneumoniae* bloodstream infections, 7 died from causes related to their underlying diseases, and 1 living patient was no longer followed at NIH. Among four patients who returned periodically for medical care, two exhibited long-term carriage of *bla*_KPC_^+^ *K. pneumoniae*; their isolates were studied further to understand possible plasmid diversification during long-term carriage.

**FIG 1:**
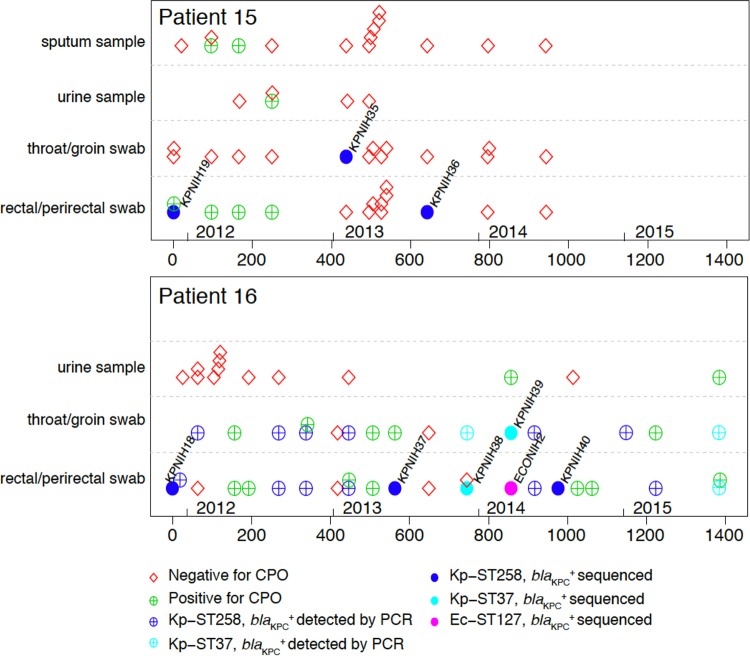
Patient carriage of carbapenemase-producing organisms, initiating with the 2011 *K. pneumoniae* outbreak strain. (Top panel) Patient 15 timeline of culture results. (Bottom panel) Patient 16 timeline of culture results. Cultures negative for carbapenemase-producing organisms (CPOs) are shown as red diamonds. Sequenced isolates from CPO-positive cultures are shown as filled circles. Other CPO-positive cultures are shown as plus signs. CPO positives are colored by organism as follows: unidentified, green; *K. pneumoniae* ST258, blue; *K. pneumoniae* ST37, cyan; *E. coli* ST127, magenta. For both the top panel and the bottom panel, CPO-negative cultures may have been positive for carbapenem-sensitive organisms. The numbers below the *x* axis represent the numbers of days since first collection of a *bla*_KPC_^+^ culture, with the years indicated above the *x* axis.

Patients 15 and 16 from the 2011 outbreak both exhibited *bla*_KPC_^+^ cultures in the years following the outbreak. Patient 15 was followed for a pulmonary condition and had six follow-up cultures in 2012 and two in 2013 that grew *bla*_KPC_^+^ *K. pneumoniae*, followed by consistently CPO-negative cultures in 2014 (Fig. 1). Patient 16 was admitted to our institution in 2011 for treatment of a fungal infection associated with an immunodeficiency. *bla*_KPC_^+^ organisms were cultured from numerous rectal/perirectal and throat/groin swabs throughout 2011 to 2015, and, as of the date of manuscript submission, patient 16 remained colonized with *bla*_KPC_^+^ *K. pneumoniae* (Fig. 1). During the course of this study, patients 15 and 16 received multiple courses of antibiotics across different classes. The only course of carbapenems in our institution was meropenem administered in the weeks before and after the initial colonization with *bla*_KPC_^+^ *K. pneumoniae.*

### Patient 15: plasmid recombination with gene duplication.

The 2011 NIH outbreak was confirmed to be clonal on the basis of nearly perfect DNA sequence identity across the 5.4-Mb genome, with 11 shared signature single-nucleotide variants (SNVs) that evolved in the index patients and in the patients who were colonized early. The 2011 *bla*_KPC_^+^ *K. pneumoniae* isolate from patient 15 (day 0; KPNIH19) has clear epidemiological and genomic connections to the 2011 clonal outbreak. Specifically, KPNIH19 shares nearly perfect sequence identity with isolates from this outbreak, including 11 shared SNVs. This first isolate from patient 15 carries the three plasmids that characterize the outbreak, namely, pKpQIL-6e6 (113 kb, carrying the *bla*_KPC_ gene), pAAC-154-a50 (15 kb), and pKPN-498 (220 kb).

In 2013, *bla*_KPC_^+^ *K. pneumoniae* isolates (KPNIH35 and KPNIH36) were detected in throat/groin and rectal/perirectal surveillance cultures from patient 15 on days 437 and 642, respectively. Both isolates were shotgun DNA sequenced and were confirmed to be sequence type 258, consistent with long-term (21-month) carriage of the outbreak strain. Moreover, KPNIH35 and KPNIH36 share nearly perfect sequence identity and the 11 signature SNVs that directly link these isolates to the 2011 outbreak. However, KPNIH35 and KPNIH36 carry small numbers of additional chromosomal SNVs (4 and 3, respectively), presumably due to continued alterations corresponding to long-term carriage. Some of these SNVs are unique to each strain, suggesting the possibility of modest genetic diversification in the different body sites of patient 15. Similar results were observed for the index patient of the 2011 outbreak, with clones identified in throat and groin that shared core SNVs but differed by 3 and 4 unique SNVs.

**FIG 2:**
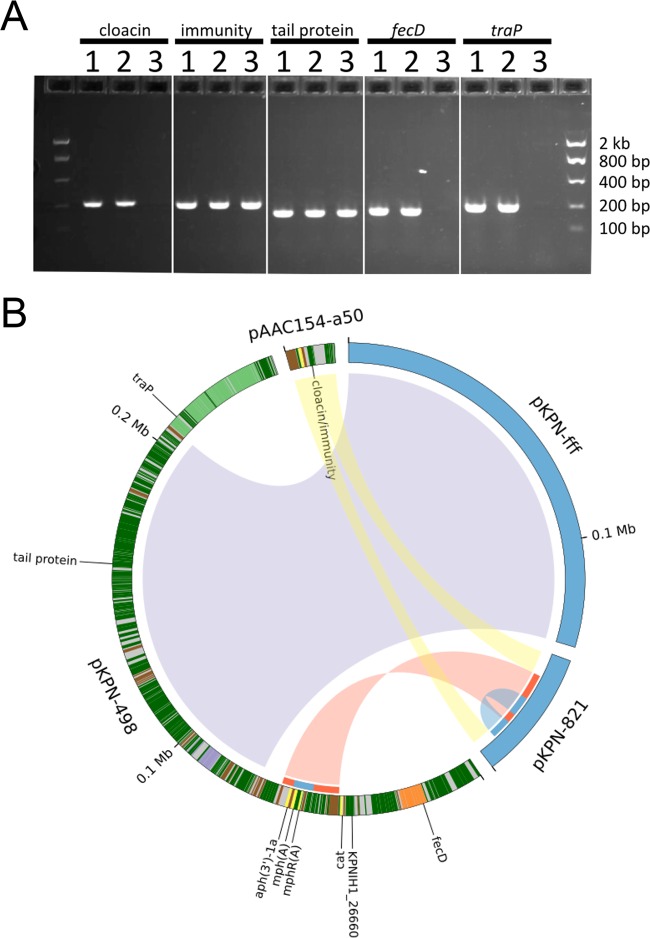
Isolates from patient 15 exhibited plasmid recombination between 2011 and 2013. (A) PCR results for marker genes. Isolates from patient 15 are numbered in order of isolation as follows: 1, day 0; 2, day 437; 3, day 642. Marker genes are indicated for each set of wells. (B) Rearrangements of the pKPN-498 and pAAC154-a50 plasmids that characterized the 2011 outbreak are indicated by ribbons connecting to the 2013 plasmids pKPN-fff and pKPN-821. Ribbons are colored for visualization purposes and do not have meaning. Gene annotations are indicated on the 2011 plasmids as follows: *tra* genes, light green; antibiotic resistance, yellow; iron acquisition, orange; metal efflux, purple; mobile elements, brown. Other genes are indicated in dark green. Select genes described in the text are labeled. The recombination/duplication event is highlighted on the inner ring, with the recombination region shown in red and the duplicated region shown in blue.

The genome assembly of KPNIH35 aligns well to the reference genome from the index patient (KPNIH1) and is consistent with continued carriage of intact copies of all three plasmids from the outbreak. However, the short-read assembly of KPNIH36 contains three plasmids with only pKpQIL intact; both pKPN-498 and pAAC154-a50 have undergone recombination. The presence of plasmid rearrangements was confirmed using targeted PCR assays that indicated that the pAAC154-a50 plasmid from KPNIH36 had lost the cloacin gene but not the nearby cloacin immunity gene (Fig. 2A). In addition, it was noted that marker genes in the iron acquisition cluster and *tra* gene locus were absent or disrupted in pKPN-498 in KPNIH36.

To precisely define the recombined genomic regions detected in KPNIH36, long-read single-molecule sequencing was performed in addition to the short-read sequencing to generate a complete genome. The fully contiguous genome confirmed that pKPN-498 genes are disrupted or deleted due to a complex plasmid recombination event that resulted in two new plasmids, pKPN-fff and pKPN-821 (Fig. 2B). Plasmid pKPN-fff is a 133-kb portion of the larger pKPN-498 plasmid. The *tra* genes, from the region flanking the excised pKPN-fff plasmid, have been lost, as confirmed by two independent sequencing methods and by targeted PCR (Fig. 2A). Similarly, the iron acquisition gene cluster and chloramphenicol resistance gene present in pKPN-498 have been lost. The genomic fragment of pKPN-498 with genes encoding resistance to aminoglycosides and macrolides recombined with the pAAC154-a50 plasmid, disrupting the cloacin gene, to form a 40.4-kb hybrid plasmid, pKPN-821. In addition, a duplication event has resulted in duplication of the macrolide resistance genes (Fig. 2B).

### Patient 16: succession and reemergence.

Patient 16 was admitted in 2011 and has received treatment at the NIH Clinical Center and at health care facilities in another state on multiple occasions since 2011. Perirectal and throat/groin swabs obtained during admission to our institution have, with few exceptions, consistently grown *bla*_KPC_^+^ *K. pneumoniae*. Data from isolates collected in 2011, 2012, and 2013 suggested continuous carriage of ST258 *bla*_KPC_^+^ *K. pneumoniae* for 563 days. From December 2013 to March 2014 (days 745 to 857), a different *bla*_KPC_^+^ *K. pneumoniae* sequence type (ST37) was isolated on three occasions. In addition, a *bla*_KPC_^+^ *E. coli* strain was isolated on day 857. In 2014 (days 916 to 1223), ST258 *bla*_KPC_^+^ *K. pneumoniae* was once again detected in multiple samples, consistent with reemergence of the outbreak strain with which the patient had by then been colonized for 4 years. In September of 2015 (day 1384), we once again detected the ST37 *bla*_KPC_^+^ *K. pneumoniae* isolates in perirectal and urine cultures. This result is consistent with the carriage of multiple KPC^+^ organisms over an extended time period.

To examine whether clinical samples obtained from patient 16 harbored a mixed population of ST37 and ST258 *bla*_KPC_^+^ *K. pneumoniae* strains, 10 isolates were selected from a prospectively obtained cultured primary specimen (day 977 postcolonization) and subjected to a diagnostic PCR assay that distinguishes between the unique capsular polysaccharide synthesis loci found in these strains. On both dates, all 10 isolates were identified with this molecular assay as ST258 and not ST37 *bla*_KPC_^+^ *K. pneumoniae* isolates (data not shown). These data would suggest that, at a given time, one of the two strains may be more dominant.

### Evidence of a complex network of plasmids (patient 16).

To unravel the complex pattern of succession and reemergence, genomic sequences of isolates from patient 16 were obtained. The first ST37 *bla*_KPC_^+^ *K. pneumoniae* and *bla*_KPC_^+^ *E. coli* isolates were subjected to both short-read Illumina and single-molecule PacBio sequencing to obtain finished chromosomal and plasmid genome sequences. Additional isolates were shotgun sequenced with Illumina, and the genome was scaffolded by alignment with the complete PacBio genomes. Similar to the pattern revealed by the analysis of data from patient 15 described above, nearly perfect chromosomal sequence identity and 11 signature SNVs link the ST258 *bla*_KPC_^+^ *K. pneumoniae* isolates (KPNIH37 and KPNIH40) collected from patient 16 to the 2011 outbreak. KPNIH37 and KPNIH40 also have a small number of unique SNVs that again suggest continued diversification among the population members.

**FIG 3:**
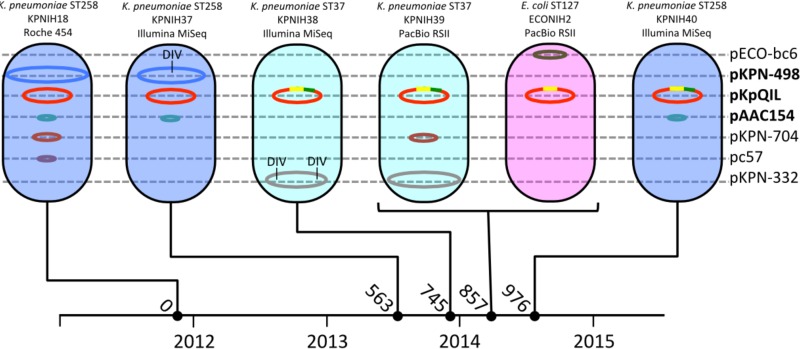
Seven plasmid backbones are associated with three microbial populations isolated from patient 16. The genus, species, and sequence type are noted above each strain illustration along with the strain name and sequencing platform. Plasmid names are listed on the right, with the 2011 outbreak strain plasmids indicated in bold. The pKpQIL recombinant regions are noted in yellow and green. DIV, deletion/insertion variants. The timeline at the bottom is labeled with years indicated below the timeline and numbers of days since first *bla*_KPC_^+^ culture indicated above the timeline.

In total, seven plasmid backbones were detected across the ST258 and ST37 *bla*_KPC_^+^ *K. pneumoniae* and *bla*_KPC_^+^ *E. coli* isolates from patient 16 (Fig. 3). Five of the seven were present in the original 2011 KPNIH18 strain isolated from patient 16. In addition to the three plasmids characterizing the outbreak strain, this patient’s 2011 isolate carried two additional small plasmids, pKPN-704 (AKAI01000038) and pc57 (AKAI01000057). The pKPN-704 plasmid is 37 kb and carries genes for 43 proteins, including a number of conjugal transfer proteins and partitioning proteins and a toxin/antitoxin addiction system. The pc57 plasmid is 12.6 kb and carries 13 genes for proteins with annotated functions involved in plasmid stability and partitioning. Neither pKPN-704 nor pc57 carries genes with obvious antibiotic resistance or virulence functions.

The six *bla*_KPC_-negative plasmids show independent patterns of inheritance and recombination. The pAAC154 plasmid is found in all three ST258 *K. pneumoniae* strains but is not present in the ST37 *K. pneumoniae* or *E. coli* isolates. pKPN-498 was found in ST258 *K. pneumoniae* isolate KPNIH37 (day 563), but as seen in patient 15, it has lost genes associated with iron acquisition and conjugation. The pKPN-498 plasmid was completely missing from ST258 *K. pneumoniae* KPNIH40 isolated on day 976. The pKPN-704 plasmid is absent from the ST258 *K. pneumoniae* isolate KPNIH37 (day 563) but was transferred to (or occurs independently in) the ST37 lineage. The pc57 plasmid does not appear to have been maintained outside the original KPNIH18 isolate from 2011. The presence or absence of these plasmids points to the independent properties controlling their inheritance and stability.

Two additional plasmids were detected that are not associated with the original outbreak strain. The ST37 *K. pneumoniae* isolate from day 857 (KPNIH39) carries a 285-kb plasmid (pKPN-332) with little similarity to published references. pKPN-332 is predicted to carry genes for resistance to arsenic, tellurium, and copper. The ST37 (KPNIH38) strain from day 745 carries a version of the pKPN-332 plasmid with two large deletions encompassing the arsenic and tellurium gene clusters. The 2014 *E. coli* isolate was found to carry a 101-kb plasmid not seen in other isolates, pECO-BC6. This plasmid is closely related (>99.9% identical) to members of a family of IncFIB/IIA plasmids that have been found to be associated with uropathogenic (pUTI89 and pEC14_114) (16, 17) and neonatal (pRS218) meningitis strains (18) of *E. coli*. An important difference is the absence of a 13.2-kb region encompassing the virulence-associated *cjrABC* locus and *senB* (*tieB*) gene, encoding putative iron uptake gene products and an enterotoxin, respectively (see Fig. S1 in the supplemental material).

### pKpQIL has undergone multiple recombinations (patient 16).

**FIG 4:**
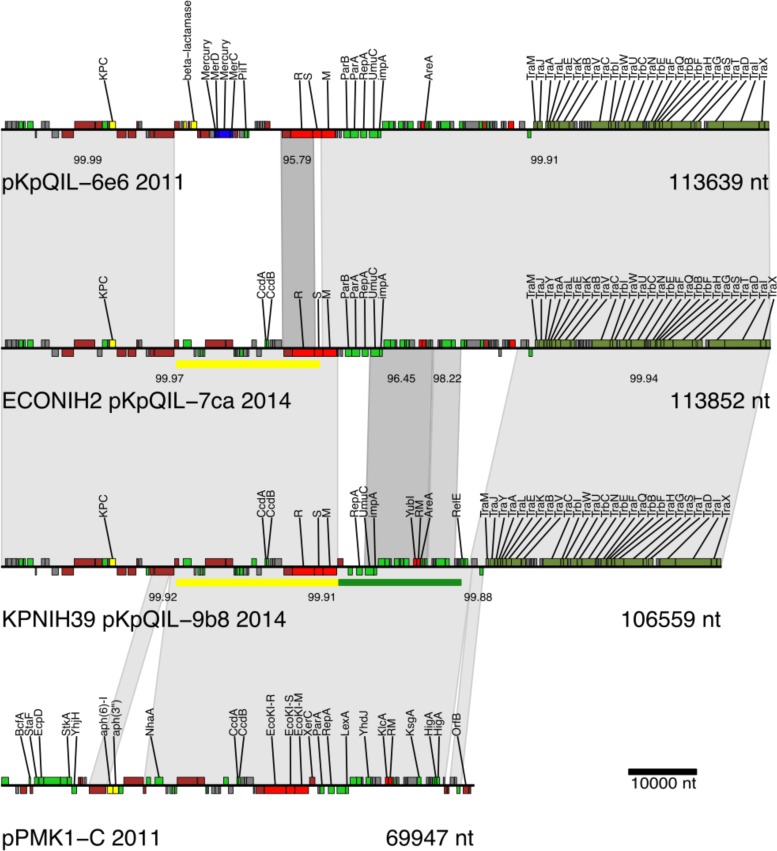
pKpQIL plasmid rearrangements from 2011 to 2014. A stacked alignment of pKpQIL and recombinant variants is shown. Genes are colored by product annotation as follows: iron-related functions are indicated in orange, copper-related functions in blue, transposes/integrases/recombinases in brown, restriction/modification genes in red (R, restriction; S, specificity; M, methylase), and conjugal transfer genes in olive green. Recombinant regions are marked by yellow and green bars. nt, nucleotides.

The pKpQIL backbone was found across all isolates that were sequenced from patient 16 and carried the *bla*_KPC_ gene, encoding a carbapenemase enzyme, in all of the isolates. Sequencing identified two recombinant versions of pKpQIL (Fig. 3 and 4). There are two explanations for these variants, if these recombinant versions arose during long-term colonization rather than being independently introduced. The first is that the variants are the results of sequential recombination events, with one or more unobserved donor plasmids resulting in a composite recombinant region. The second is that the variants are the results of two independent recombination events. It is not possible to differentiate these two scenarios, as the intermediates were likely present in uncharacterized components of the gut commensal flora and may have been only transient members of this patient’s gastrointestinal community. However, the composite recombinant region is 99.9% identical to a region of the *K. pneumoniae* pPMK1-C plasmid identified in 2011 (19), so it is possible that pPMK1-C or a similar plasmid is the unobserved donor plasmid. These recombinations result in the loss of a mercury resistance cassette and replacement of a type I restriction modification system.

## DISCUSSION

This report is one of the first to provide a high-resolution view of long-term carriage of antibiotic-resistant bacteria from the perspective of the bacterial genome, specifically, the constellation of plasmids. We demonstrated that plasmids are dynamic, readily recombining to form hybrid plasmids. Large-scale plasmid rearrangements have been reported previously in large strain collections (20, 21) but not in the context of long-term colonization of an individual patient. While this level of plasticity is not unexpected, given the natural diversity of plasmids and recombination events observed in the laboratory, this is one of the first examples of a study of long-term carriage in patients to observe these changes *in vivo*.

In both patient 15 and patient 16, the pKPN-498 plasmid was observed to be undergoing gene loss and in one case appears to have been lost from the ST258 *bla*_KPC_^+^ *K. pneumoniae* strain. Because there is no epidemiological evidence supporting the idea of a second transmission event between patient 15 and 16, this would be consistent with selective pressure serving to reduce the size of the large pKPN-498 plasmid during host adaptation. A careful review of the sequencing data from the 2011 outbreak samples identified a third case of gene loss (*fecD* marker) from pKPN-498 in patient 18 (KPNIH23; see Fig. S2 in the supplemental material). It is possible that the genes carried by pKPN-498 confer a selective advantage only while establishing colonization or for survival in the hospital environment.

The *bla*_KPC_-carrying pKpQIL plasmid was stable across the course of the 2011 outbreak and the entire long-term colonization of patient 15. However, for patient 16, two recombination events, either sequential or parallel, resulted in the detection of variant plasmids in both *bla*_KPC_^+^ *K. pneumoniae* and *bla*_KPC_^+^ *E. coli* that are predicted to have new properties. Compared to the reference pKpQIL plasmid from 2006 (NC_014016), both variants lost a mercury resistance locus and replaced the type I restriction enzyme. The replacement of the restriction modification system between the original and the variant pKpQIL plasmids results in the presence of DNA sequence recognition subunits with only 38% identity and 51% similarity at the amino acid level, and this could have an impact on host range. The pKpQIL plasmid has been detected previously in *E. coli* (20), and, in that case, the entire restriction modification system was part of a 14.5-kb deletion.

This study had a number of limitations and caveats. The first was that isolates were collected using a surveillance protocol focused on supporting patient care. For that reason, the study was subject to sampling bias because it is not clinically practical to isolate and store multiple isolates of the same morphology, as would be required to survey the bacterial population at a given time point. On one occasion, while streaking out the KPNIH36 isolate frozen stock, we were able to detect two different morphologies of *K. pneumoniae*, described as consisting of rare convex colonies and many flat colonies. Attempts to repeat this were not successful, indicating that the convex colonies are rare. While the KPNIH36 isolate contains the recombined pKPN-821 and pKPN-fff plasmids, PCR assays show that these rare convex colonies are genetically different and carry intact pKPN-498 and pAAC154 plasmids, indicating the continued presence of the original outbreak strain. In addition, the pattern of loss and reemergence seen for the ST258 and ST37 *K. pneumoniae* strains in patient 16 indicates that there is likely mixed colonization, competition between strains of *K. pneumoniae*, and reemergence of previously detected strains. Future experiments should include PCR panels or metagenomic sequencing to address the entire population of organisms in a sample. Genetic variation within a single clinical sample has been observed previously in *Burkholderia dolosa* from individuals with cystic fibrosis (22). It was shown in that study that *B. dolosa* exhibited a “diverse community” rather than corresponding to a “dominant lineage” model.

The selection for *bla*_KPC_^+^ organisms was a second limitation of this study. Antibiotic-resistant bacteria typically exist in a complex community of microbes that are, for practical purposes, invisible due to factors such as fastidious growth conditions or antibiotic sensitivity. Those community members are potentially a rich source of plasmids and genetic diversity.

From a technical perspective, this study leveraged data from a wide variety of sequencing platforms. The earliest genomes analyzed in this study were sequenced with the Roche 454 platform, but the bulk of microbial sequencing utilizes genome assemblies from the Illumina platform. For this study, we also constructed finished genomes on a PacBio instrument to serve as references upon which to scaffold shotgun sequence data from the other isolates. For these experiments, PacBio and Illumina datasets were obtained from the same isolates to serve as internal controls and to benchmark the two sequencing platforms. Finally, marker gene-based PCRs were used to screen for plasmids and variants across a large number of unsequenced isolates. Overall, most of the details observed in this study were first detected in the short-read data, demonstrating the value of this widespread technology. That said, fully contiguous genomes, such as those produced by the PacBio sequencing platform, are invaluable for dissecting the details of plasmid recombination and to provide references for future studies.

## MATERIALS AND METHODS

### Clinical ascertainment.

The NIH Clinical Center conducts extensive microbial surveillance for CPO carriage, including collection of perirectal swabs on admission for nearly all patients and twice weekly among patients occupying high-risk wards, as described by Conlan et al. (5).

### Genome sequencing and molecular assays.

Genomic DNA was prepared from bacterial isolates grown overnight on blood agar. DNA was extracted using a Promega Maxwell 16 nucleic acid purification system with a tissue DNA purification kit. Genomic DNA was subjected to further RNase treatment and subsequent cleanup using a Zymo Research Genomic DNA Clean and Concentrator kit (D4010). This genomic DNA was used to prepare libraries for both Illumina and single-molecule real-time (SMRT) sequencing. Illumina libraries were created for all isolates using Nextera library chemistry and were then sequenced on an Illumina MiSeq instrument. The resulting paired-end reads were assembled using SPAdes version 3.5.1 (23) and error corrected using Pilon (24). Select genomes were also sequenced using SMRT sequencing. Libraries were constructed using a SMRTbell template kit, ver 1.0. The DNA was size selected for the range 7 kb to 50 kb using a BluePippin platform with a 0.75% gel cassette. Sequencing was performed on a PacBio RSII instrument using P5 polymerase binding and C3 sequencing kits with magnetic bead loading and 180-min acquisition. Genome assemblies were performed using HGAP and Quiver as part of SMRTAnalysis, version 2.3. For every genome completed on the PacBio instrument, we also produced an independent genome sequence on the Illumina instrument both to resolve any sequencing errors and to use as a model for aligning short reads to the reference PacBio genome.

PCR assays were designed based on genomic data using Primer3 (25) (see Table S1 in the supplemental material). PCRs used TaKaRa LA HS Taq (RR042B) and a 400 nM concentration of each primer. Reaction mixtures were denatured at 95°C for 3 min and amplified for 30 cycles of 95°C for 20 s, 58°C for 30 s, and 72°C for 1 min.

### Plasmid reconstruction from short-read WGS data.

Plasmids were reconstructed from whole-genome shotgun (WGS) data by scaffolding using complete reference plasmids and ABACAS (26). In some cases, scaffolding of WGS data indicated that a plasmid had a deletion. Deletions were verified *in silico* by confirming that the unassembled reads did not map to that region. Deleted regions were further tested by designing primers corresponding to genes in the deleted regions and performing PCR. For KPNIH18, scaffolding resulted in the identification of two contigs that could not be incorporated into the expected chromosome or plasmid references. These were predicted to be plasmids on the basis of the presence of plasmid-associated genes and greater than 3-fold genomic copy numbers. One contig (AKAI01000038) was later detected as a circular plasmid (pKPN-704) in a different strain. The other contig (AKAI01000057) was never detected in another strain but was confirmed to be circular by a PCR designed to span the contig ends. In both cases, PCR assays were designed using marker genes to verify the absence of these small plasmids in the reported strains.

### Plasmid Inc group typing.

Plasmid incompatibility (Inc) groups were assigned by one or more methods, including the following: analysis of similarity to published plasmids, *in silico* PCR with typing primers from Caratolli et al. (27), pMLST typing (http://pubmlst.org), and use of the PlasmidFinder v 1.3 tool (28).

### Nucleotide sequence accession numbers.

Sequences determined in this work have been deposited in GenBank under accession numbers CP008827.1, CP008830.1, CP008828.1, CP008829.1, AKAJ00000000, LRIM00000000, CP014647, CP014650, CP014648, CP014649, AKAI00000000, LRRE00000000, LTBD00000000, CP014762, CP014765, CP014764, CP014763, CP014667, CP014669, CP014668, LTDV00000000, and AKAN00000000.

## SUPPLEMENTAL MATERIAL

Figure S1  Stacked alignment of pECO-BC 6 (this study) to pUTI89 (CP000244) and pEC_114 (GQ398086). Genes are colored by product annotation as follows: iron-related functions are indicated in orange, copper-related functions in blue, transposes/integrases/recombinases in brown, and conjugal transfer genes in olive green. All other protein-coding genes are indicated in light green. Gray alignment bands are labeled with the percent identity between the indicated segments. Download Figure S1, PDF file, 0.03 MB

Figure S2  Stacked alignment of pKPN-498 (from 2011 outbreak strain) to deletion variants found in isolates from patients 18, 16, and 15. The plasmids from patient 18 and 16 are shown as scaffolds, with contig joins marked in red. Genes are colored by product annotation as follows: iron-related functions are indicated in orange, copper-related functions in blue, transposes/integrases/recombinases in brown, and conjugal transfer genes in olive green. All other protein-coding genes are indicated in light green. Gray alignment bands are labeled with the percent identity between the indicated segments. Download Figure S2, PDF file, 0.04 MB

Table S1  Primers. Table S1, PDF file, 0.05 MB

Table S2  Summary of genomic data. Table S2, PDF file, 0.04 MB
