# Supplementary figures and images for: Plasmid Dynamics in KPC-Positive Klebsiella pneumoniae during Long-Term Patient Colonization
Source: mBio. 2016 Jun 28;7(3):e00742-16. doi: 10.1128/mBio.00742-16 (PMC4937214; doi:10.1128/mBio.00742-16)

# Supp 1

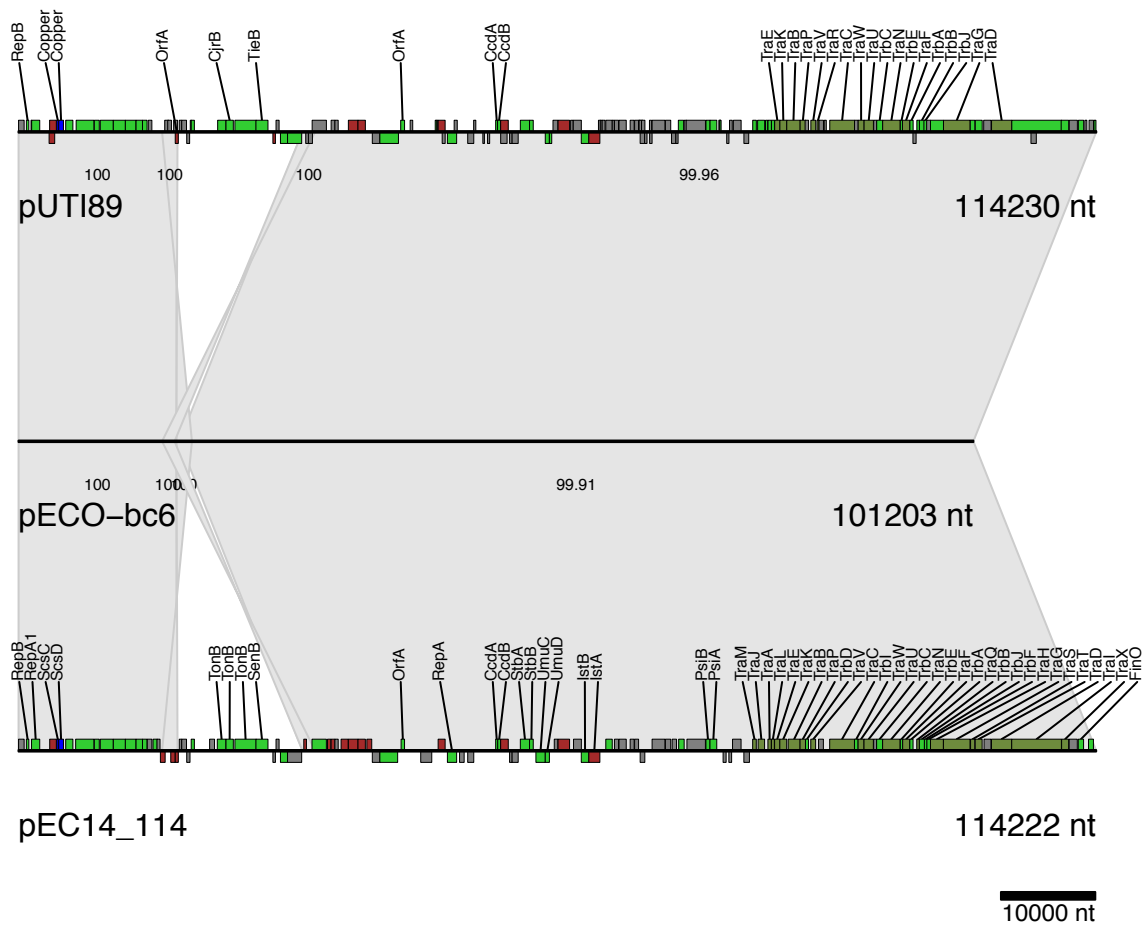

Supplement: Figure S1 — Stacked alignment of pECO-BC 6 (this study) to pUTI89 (CP000244) and pEC_114 (GQ398086). Genes are colored by product annotation as follows: iron-related functions are indicated in orange, copper-related functions in blue, transposes/integrases/recombinases in brown, and conjugal transfer genes in olive green. All other protein-coding genes are indicated in light green. Gray alignment bands are labeled with the percent identity between the indicated segments. Download [file mbo003162868sf1.pdf]

# Supp 2

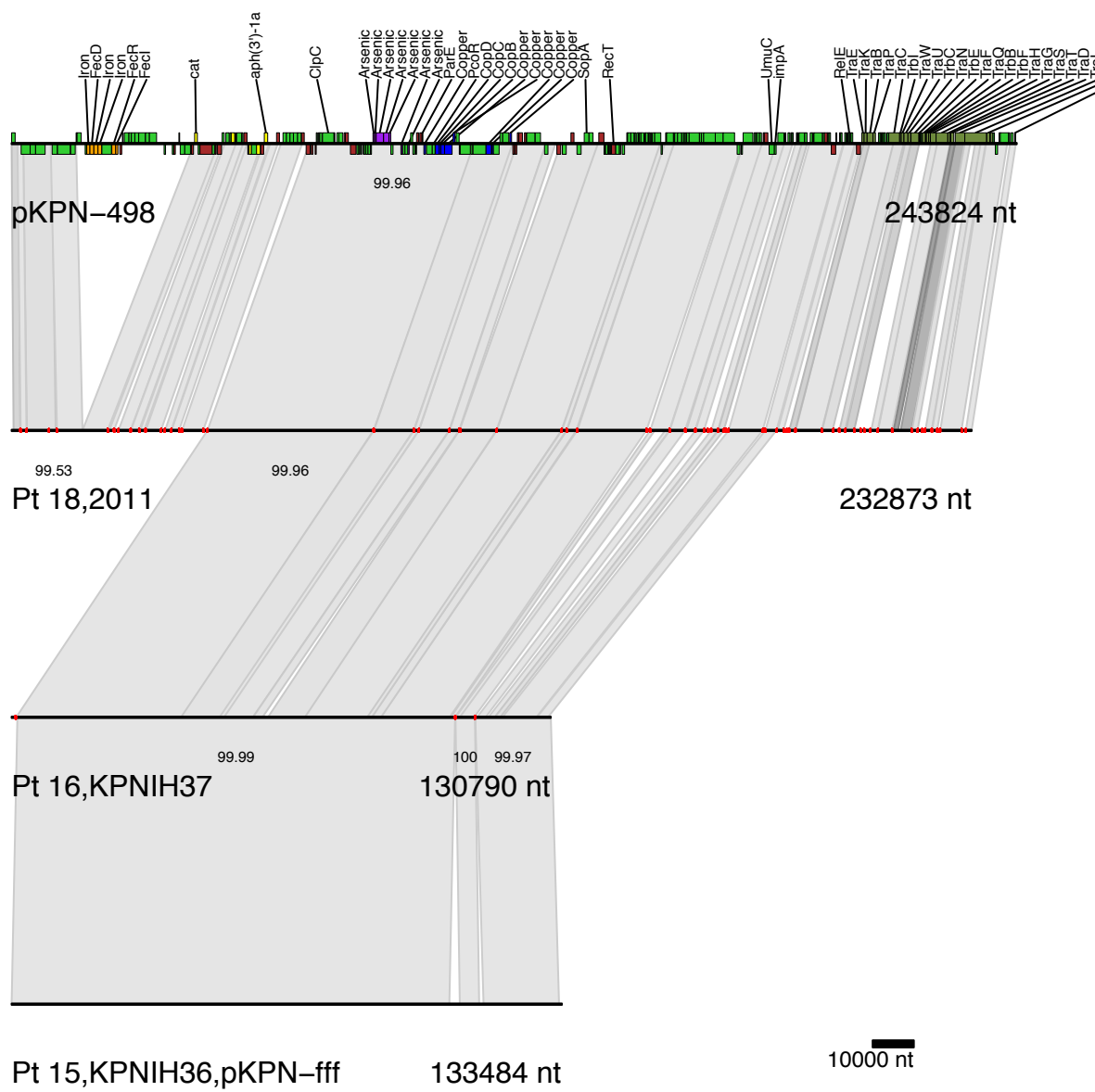

Supplement: Figure S2 — Stacked alignment of pKPN-498 (from 2011 outbreak strain) to deletion variants found in isolates from patients 18, 16, and 15. The plasmids from patient 18 and 16 are shown as scaffolds, with contig joins marked in red. Genes are colored by product annotation as follows: iron-related functions are indicated in orange, copper-related functions in blue, transposes/integrases/recombinases in brown, and conjugal transfer genes in olive green. All other protein-coding genes are indicated in light green. Gray alignment bands are labeled with the percent identity between the indicated segments. Download [file mbo003162868sf2.pdf]
