# Supplementary material for: Plasmid Dynamics in KPC-Positive Klebsiella pneumoniae during Long-Term Patient Colonization
Source: mBio. 2016 Jun 28;7(3):e00742-16. doi: 10.1128/mBio.00742-16 (PMC4937214; doi:10.1128/mBio.00742-16)
Supplement: Table S1 — Primers. [file mbo003162868st1.pdf]

Supplemental Table 1 - Primers

Patient 15 Plasmid Recombination Markers

Sequence	Target Gene	Name	Forward	Reverse
pAAC154	cloacin	CRE17	AAACGTGTGGCGGATATTGT	CGGACGTGAATCACCTTTTT
pAAC154	immunity	CRE18	GAAAGTCTGGGGATGCCTTT	AAAAACCCCGGTATTCATCC
pKPN-498	fecD	CRE65	CTGTGCGCTGTTATCACTCC	TACGCACAATCCCCTGTACC
pKPN-498	phage tail protein	CRE59	ACACCTGTTTCCGCAAATTC	TGTTGAGCTTGGCAGTATCG
pKPN-498	TraP	CRE68	AGACTGCACCACCGTCATTA	AGATCGGGCTAGAATCAGCC

Patient 16 pKpQIL Recombination Markers

Sequence	Target Gene	Name	Forward	Reverse
pKpQIL	QIL_TE37	CRE50	CGACAGATAAATCCGCTGCT	CACCGCTTTAGGCACATTTT
pKpQIL	QIL_merc	CRE51	TCGTCAGGTAGGGGAACAAC	GGCTTCATCAAACTGGTGGT
pKpQIL	QIL_nha	CRE52	CTTGACCTCATCAACGAGCA	GCATGACGATGAATGACCTG
pKpQIL	QIL_sos	CRE53	GTTCCCTTGTTACCCTGCAA	GCTTCTTCCACCTGCTTCAG
pKpQIL	QIL_relE	CRE54	AAAAATGGCCAGGCTACTCA	TTTCCTCCAGTCTGCGAAGT

Patient 16 Capsular Polysaccharide Sysnthesis Locus Markers

Sequence	Target Gene	Name	Forward	Reverse
ST258 Chromosome	RfbN_19637	CRE61	TTGCTGAGGAAGCTGGTTTT	AGGCAATTGTCGACCGTAAG
ST258 Chromosome	rfbF_19637	CRE62	AATCCCGATGATGGACTTGA	CCATCGCCTTGGTATGATAAA
ST37 Chromosome	wzy_21757	CRE63	CTTCCGCCATATGGTGACTC	ACCCCGAAAGTTCACAATCA
ST37 Chromosome	wzx_21757	CRE64	GTCGCCGTTAGTCTGGCTAC	CGAGATGACGACACACCCTA

Patient 16 Small Plasmid Markers

Sequence	Target Gene	Name	Forward	Reverse
pc57	TraG	CRE55F	ATGGCTCCGGTAAGGGTATC	GAACGGGTCCACCAGATAGA
pc57	rep	CRE56F	TGGTGTTGTCCGAAGGCTAT	TTGTATTCTTGCGCGAACTG
pc38/pKPN-704	Primase	CRE57F	GACCGCTGACAATGCCTATT	TCTGTGCCAGGTATCAGCAG
pc38/pKPN-704	Mpr	CRE58F	AGACGAAATCGCCATGAATC	GCATAGTCGGCCATCTTCTC
