# Supplementary material for: Plasmid Dynamics in KPC-Positive Klebsiella pneumoniae during Long-Term Patient Colonization
Source: mBio. 2016 Jun 28;7(3):e00742-16. doi: 10.1128/mBio.00742-16 (PMC4937214; doi:10.1128/mBio.00742-16)
Supplement: Table S2 — Summary of genomic data. [file mbo003162868st2.pdf]

Supplemental Table 2 - Summary of Genomic Data

Patient ID	Strain	Platform	BioProject	BioSample	Accession	Sequence	MLST	Incompatibility group(s) [1,2]
1	KPNIH1	PacBio	PRJNA73191	SAMN01057611	CP008827.1	chromosome	ST258	n.a.
					CP008830.1	pKpQIL-6e6	n.a.	IncFII/FIB
					CP008828.1	pAAC154-a50	n.a.	ColE1
					CP008829.1	pKPN-498	n.a.	IncFII/FIB
15	KPNIH19	gs454	PRJNA80233	SAMN01057638	AKAJ00000000	WGS	ST258	IncFII/IncFIB/ColE1
	KPNIH35	MiSeq	PRJNA303003	SAMN04287036	LRIM00000000	WGS	ST258	IncFII/IncFIB/ColE1
	KPNIH36	PacBio	PRJNA284365	SAMN03701676	CP014647	chromosome	ST258	n.a.
					CP014650	pKpQIL-6e6	n.a.	IncFII/FIB
					CP014648	pKPN-821	n.a.	ColE1
					CP014649	pKPN-fff	n.a.	IncFII
16	KPNIH18	gs454	PRJNA80231	SAMN01057635	AKAI00000000	WGS	ST258	IncFII/IncFIB/ColE1
	KPNIH37	MiSeq	PRJNA279671	SAMN03455991	LRRE00000000	WGS	ST258	IncFII/IncFIB/ColE1
	KPNIH38	MiSeq	PRJNA279672	SAMN03455992	LTBD00000000	WGS	ST37	IncFII
	KPNIH39	PacBio	PRJNA279611	SAMN03455930	CP014762	chromosome	ST37	n.a.
					CP014765	pKpQIL-9b8	n.a.	IncFII/FIB
					CP014764	pKPN-704	n.a.	non-typable
					CP014763	pKPN-332	n.a.	non-typable
	ECONIH2	PacBio	PRJNA279612	SAMN03455931	CP014667	chromosome	ST127	n.a.
					CP014669	pKpQIL-571	n.a.	IncFII/FIB
					CP014668	pECO-bc6	n.a.	IncFII/FIB
	KPNIH40	MiSeq	PRJNA279619	SAMN03455939	LTDV00000000	WGS	ST258	IncFII/ColE1
18	KPNIH23	gs454	PRJNA156865	SAMN01057657	AKAN00000000	WGS	ST258	IncFII/IncFIB/ColE1

(1) Carattoli A, Bertini A, Villa L, Falbo V, Hopkins KL, Threlfall EJ. J Microbiol Methods. 2005 Dec;63(3):219-28. Epub 2005 Jun 2.
(2) Carattoli A, Zankari E, García-Fernández A, Voldby Larsen M, Lund O, Villa L, Møller Aarestrup F, Hasman H4. AAC. 2014 Jul;58(7):3895-903.
